# Social cohesion, social trust, social participation and sexual behaviors of adolescents in rural Tanzania

**DOI:** 10.1186/s12889-019-6428-7

**Published:** 2019-02-14

**Authors:** Albino Kalolo, Jacob Mazalale, Anja Krumeich, Michelene Chenault

**Affiliations:** 1 Department of Public Health, St. Francis University College of Health and Allied Sciences, P.O.Box 175, Ifakara, Tanzania; 2 Department of Economics, University of Malawi, Chancellor College, P. O Box, 280 Zomba, Malawi; 3 Faculty of Health, Medicine and Life Sciences, Department of Health, Ethics and Society, Maastricht University, Maastricht, The Netherlands

**Keywords:** Social cohesion, Social trust, Social participation, Sexual behaviors, Adolescents, Rural Tanzania

## Abstract

**Background:**

Social cohesion, defined as a glue holding society together, has been found to influence several aspects of human behavior. Social cohesion, being composed of social trust and social participation, is a social factor that may influence sexual behaviors. Unfortunately, studies investigating the influence of social cohesion on sexual behaviors among young people are scarce. This study examined the influence of social cohesion on safe sexual behavior among adolescents in rural Tanzania.

**Methods:**

A cross-sectional study was conducted among 403 school adolescents of the Newala district, between May and August 2010. Socio-demographic characteristics, social cohesion (social trust and social participation) and sexual behavior (age at sexual debut, intention to use and reported condom use, number of sexual partners) were obtained through self-administered questionnaires**.** Data analysis was performed using descriptive statistics and binary logistic regression.

**Results:**

Sexual debut at under 13 years of age was reported by 12% of the respondent. A majority (71%) reported multiple sexual partnerships and half of the participants reported to have used a condom at their last sexual encounter. The intention to use a condom was reported by 77% of the respondents. Having multiple sexual partnerships was associated with social trust only (odds ratio: 3.5, 95% CI 1.01–12.3) whereas reported condom use was related with social cohesion (odds ratio 4.8 95% CI 1.66–14.06). Social cohesion, trust or participation was not associated with young age at sexual debut or intention to use a condom. Being a female (odds ratio 2.07 95% CI 1.04–4.12.) was associated with intention to use a condom.

**Conclusion:**

This study indicates that social cohesion and socio-demographic factors influence actual behavior performance and behavioral intentions. The findings point to the importance of collecting more evidence on social cohesion and sexual behaviors in different settings and designing interventions that enhance social cohesion among adolescents in order to reinforce positive sexual behaviors.

**Electronic supplementary material:**

The online version of this article (10.1186/s12889-019-6428-7) contains supplementary material, which is available to authorized users.

## Background

Involvement in ‘unsafe’ sexual behaviors such as, unprotected sex, multiple or concurrent sex partners, is associated with undesired consequences like acquiring sexually transmitted infections, human immunodeficiency virus (HIV), and acquired immunodeficiency syndrome (AIDS). Particularly in Sub-Saharan Africa (SSA), unsafe sexual behavior among adolescents has been a persistent obstacle in the fight against the spread of HIV/AIDS. Recent evidence shows that SSA accounts for 70% of the global burden of new HIV infections. Moreover, the substantial increase in unsafe sex behavior might counteract gains made in combating HIV/AIDS on the continent [1–4].

Tanzania is one of the sub-Saharan African countries with a high burden of HIV/ AIDS [5–8]. The thirty years history of the HIV/AIDS in Tanzania has been characterized by a generalized epidemic, with young people being at the center of new infections. In this period, while there have been a number of interventions to promote safe sex behaviors, there has been inequality in to what extent these interventions reach adolescents [5, 8–11]. These inequalities are characterized by varying demographics, such as rural-urban and in-school-out of school differences.

Since 2005, the number of in-school adolescents in Tanzania has increased dramatically following country-wide education sector reforms that required district authorities to construct secondary schools and enroll students in each ward of a district [12, 13]. This reform created more opportunities for adolescents to join secondary level education. This reform could be seen as an opportunity to introduce and scale up sexuality education and safe sex promotion for adolescents in the school environment. Many adolescents, who would otherwise have been out of school, are now enrolled in schools where they can easily be reached by in-school interventions. The school environment offers an ideal setting and infrastructure to support the promotion of safe sex behaviors among adolescents. Evidence has been given that the school environment is a better place for health promotion due to high social cohesion within the cultural context in which the school is located [14–16].

The concept of social cohesion, although complex, embodies a theoretical underpinning that is becoming an important analytical tool of various human behaviors experienced by individuals in certain social contexts. Social cohesion, together with its dynamics, is multidimensional as explained by the multiplicity of available frameworks and approaches used to study it. Existing literature defines social cohesion in different ways: 1) the capacity of societies, not merely groups or networks, to manage collective action and solve problems [17]; 2) the glue that holds the society together [18]; 3) social climate characteristics of the society and surrounding environment [19]; 4) absence of social exclusion, interaction and connectedness based on social capital, shared values, and community interpretations based on group identity [20]. 4) Property which the whole society and individuals within are bound together through the actions of specific attitudes, behaviors, rules, and institutions which rely on consensus rather than coercion [21]. We draw on definitions from Woolcock [17] and Janmaat [18] where social capital is regarded as a prerequisite for social cohesion [22] . In line with previous literature, we view social capital as sacrifice (time, effort, and consumption) made by individuals to cooperate with others [23, 24]. In this study we consider social cohesion as a characteristic of a society that depends on accumulated social capital [23, 24].

Sexual behaviors of adolescents of sub-Saharan Africa have been extensively studied and interventions that address the various determinants of sexual behaviors have been implemented [3, 10, 25, 26, 27]. The emphasis across studies has been on risk and protective factors that act at individual and interpersonal levels with limited inference on wider social ecological determinants. The contextual issues related to how the society is organized, and the level of cohesiveness at societal level, remains widely unexplored. It is important to further explore social cohesion due to the substantial existing evidence that shows social cohesion provides an avenue for health promotion, makes the society feel connected, and provides group level influence that facilitates the organization of community activities to address issues that affect the society [21].

The relationship between social cohesion and sexual behaviors in other groups such as sexual workers [28], adults [29] or outside sub-Saharan Africa [30] has been investigated. However, studies that exclusively focus on the link between social cohesion and sexual behaviors among school adolescents are scarce in the SSA settings. Of particular interest are adolescents living in rural areas with limited access to social marketing programs and the media.

We draw on the social theories [27, 31–34] to understand the link between social cohesion and sexual behaviors within the context of reduction of new HIV infections among young people. The theories, among other things, posit a view that behaviors happen within the context of social interactions and experiences.

In this study, we investigate whether social cohesion has influence on sexual behaviors of school adolescents within the context of reducing new HIV infections among rural school adolescents. This research arose because it is clear that when adolescents join secondary schools they are exposed to new social networks, which can have varying effects on perceptions and experiences regarding sexuality issues. We hypothesized that social cohesion will be associated with adolescents’ intentions to engage in safe sex and safe sex practices. The study addressed three questions: 1) To what extent did school adolescents practice safe sex behaviors? 2) To what extent did social cohesion (social trust and social participation) and socio-demographic characteristics influence safe sex behaviors of adolescents? 3) Which social cohesion aspects were more important in enhancing safe sexual behaviors?

## Methods

### Study design and study area

This study was conducted from May to August 2010, in the Newala district in Mtwara region of Tanzania. Newala district is considered a rural location, approximately 145 km from the regional capital, bordering Mozambique in the South. As of 2010, the district had three geographical divisions (Newala, Kitangari and Chilangala), 21 geographical wards, and 155 villages. Approximately 80% of the inhabitants live outside of the district capital. The majority of inhabitants belong to the Makonde tribe, are Muslim, and predominantly speak Kimakonde and Kiswahili. Residents of Newala are mostly farmers, with the major cash crops being cashew nuts, sesame seeds, and cassava, and staple crops include cassava, maize, sorghum, and legumes. In addition, inhabitants commonly raise goats, sheep, chickens, and cows. The district population was 205,492, in 2012 [35]. Schools are distributed throughout the district whereby each village has at least one primary school and each ward with at least one secondary school. The district had a total of 117 primary schools and one special center for handicapped children in Luchingu village. The total number of primary school pupils was 44,260; 22,176 boys and 22,088 girls. The district had 26 secondary schools with an estimated total of 8169 students. The prevalence of HIV infection stood at 3.1% (antenatal care prevalence as of 2010). With regard to health care, Newala had one district hospital, three health centers, and 24 dispensaries scattered throughout the district. Almost all health facilities, except one health center, were government owned.

### Study design, population and sampling procedures

A cross-sectional study was conducted in the study area and used a multistage cluster sampling procedure to recruit the participants. Initially, the sampling frame was all ordinary level secondary schools, whereby 26 schools were identified, thereafter; three schools from each of the three geographical divisions were randomly selected, i.e., a total of nine schools were selected. These schools had a total of 4392 students with 2126 males and 2266 females. In the second step, the sampling frame consisted of all classes in each selected school; therefore, a sample of classes was obtained. All students in the selected classes were invited to participate in the study. A total of 403 participants were considered adequate to perform statistical analyses adopted in this study [36, 37] but also exceeded the calculated sample size of 389 students, with an assumption of 80% power, 42% of youth who use condoms in previous studies [5] and an estimated error margin of 0.05. All study participants were between 14 and 19 years of age.

### Data collection procedures

Primary data was collected using a pre-tested self-administered questionnaire (Additional file 1). There were three response formats for the questionnaire items: yes/ no and likert scale questions with responses ranging from three (agree, uncertain, disagree) or five categories (totally agree, agree, uncertain, disagree, and totally disagree) responses. The questionnaire was administered in the classroom, and was anonymous and voluntary; students were allowed to withdraw at any point without being questioned. After introducing the research assistants to their students, the teachers left their classroom to ensure the students’ freedom and privacy.

The training of the research assistants, and a pre-testing of the instruments, occurred prior to actual data collection. The questionnaire was translated from English to Kiswahili (the national language of Tanzania).

### Analytical approach

#### Measures

##### Outcome variables

There were two outcome variables considered: (1) intention to engage in safe sex, that is, intention to use condoms during intercourse in the future and (2) reported actual safe sex behavior (for the sexually active youth), i.e., reported condom use. For intention to engage in safe sex, the variable was defined as binary with 0 = not intending or not sure if safety precautions during their next sexual encounter i.e. condom use, and 1 = if participants intend to engage in safe sex, i.e. condom use. Three outcome variables captured sexual experience namely: age at sexual debut, number of sexual partners in the past twelve months and use of condom during the last sexual encounter. Early sexual debut was binary with 1 = started sex at young age (≤ 13 years of age), or 0 = for otherwise. More than one of sexual partner was binary with 1 = more than one partner, or 0 = otherwise. Condom use during last sexual encounter was as binary with 1 = did not use condom, or 0 = used condom.

##### Measuring social cohesion

Social cohesion is the explanatory variable for this study, and was measured as an aggregate variable of individual level social capital variables. We considered social capital to be composed of i) social trust and compliance to significant others ii) social participation. Social trust and compliance to significant others was measured by asking participants on their agreement or disagreement with statements related to trust and compliance to significant others (peers, parents, teachers and religious leaders). Exploratory factor analysis was applied to determine unidimensionality, and consistence was determined by Cronbach’s alpha for the social trust scale.

Social participation was measured through questions pertaining to students participation in safe sex promoting groups (membership in peer education groups, sports and games) in and out of the school environment (membership in youth clubs, civil society organization, traditional dance groups, sports bonanza).

The social participation at school variable was divided into five quintile categories. This was defined as an index variable that was generated using multiple correspondence analyses. Multiple correspondence analysis (MCA), an extension of correspondence analysis, allows investigation of patterns of relationships of several categorical dependent variables [38]. It is sometimes considered as a generalization of principal component analysis (PCA) for categorical variables. A similar index was constructed using PCA and the two indices had a correlation coefficient of rho> 0.99. All the variables used to build the index were binary. These included the variables: student participates in planning life skills activities as school (1 = yes participates, or 0 = otherwise), student facilitates life skills training and planning activities for the school (1 = yes facilitates, or 0 = otherwise), student acts as a role model in life skills activities (1 = yes acts, or 0 = otherwise), and student listens to HIV/AIDS messages during life skills activities at school (1 = yes listens, or 0 = otherwise).

The social participation at the community level variable was also an index with five quintiles. It was also generated using MCA. The PCA alternative has a correlation coefficient of rho> 0.98. Social participation at the community level was an index variable generated after an MCA of the following binary variables: whether the student is a member of a youth civil society organization (CSO) (1 = yes if member, or 0 = otherwise), whether the student is a member of any school or community youth group (1 = yes if member, or 0 = otherwise), whether the student is a member of a youth club or a camp dealing with HIV and AIDS issues (1 = yes if member, or 0 = otherwise), whether the student is a member a youth sports club (1 = yes if member, or 0 = otherwise), whether the student is a member of a ‘people Living with HIV and AIDS group (1 = yes if member, or 0 = otherwise), and whether the student is a member of an arts group that deals with issues concerning HIV and AIDS (1 = yes if member, or 0 = otherwise). In addition to school and community cohesion, we included age, sex, and religion as control variables.

Social cohesion was computed as a sum of scores of social trust and social participation scales.

#### Relationship of social cohesion variables and sexual behaviors

Chi square and logistic regression analysis was used to assess the association of social cohesion variables to sexual behaviors. This included separately assessing social trust and social participation, as well as assessing social cohesion as a composite variable emanating from social trust and social participation. Clustering at school level was accounted for using robust standard errors. Robust estimation is used to approximately estimate standard errors, in the presence of clustering, by correcting variance-covariance estimates.

## Results

A total of 403 adolescents participated in the study (response rate = 90%). These included 203 (50.4%) female and 200 (49.6%) male adolescents. Among the students who participated in the study, 351 (87%) were Muslim, and their age ranged from 14 to 19 years. Two hundred seventy- six (68%) of the students were within the age range of 17–19 years. There were 247 (62%) students whose schools provided training on life skills for HIV and AIDS prevention. Of these, only 13 (5%) had the strongest school participation by participating in most of the activities that were offered. In the community, 71 students (18%) had a strong social participation at the community level by participating in several HIV and AIDS related activities that were available in the community. Table 1 presents the descriptive statistics in further detail.

**Table 1:** Univariate description of the study sample (*N* = 403)

	Variable	*n*	%
Social participation at school (*n* = 247)^a^			
Weak coherent		53	21
2		51	21
3		50	20
4		80	32
Strongest coherent		13	5
Social participation at the community (*n* = 403)			
Weak coherent		122	30
2		40	10
3		81	20
4		89	22
Strongest coherent		71	18
Age (*n* = 403)			
14–16 years old		127	32
17–19 years old		276	68
Religion (*n* = 403)			
Non Muslim		52	13
Muslim		351	87
Sex (*n* = 403)			
Male		203	50
Female		200	50
Overall cohesion			
Weak coherent		92	23
2		74	18
3		76	19
4		81	20
Strongest coherent		80	20

^a^Please note that 247 were going to schools which provided training on life skills for HIV and AIDS prevention

Table 2 presents the outcome variables, i.e. sexual behaviors observed among the students. 12% (*n* = 49) had their age at sexual debut at 13 or less years old and 71% (*n* = 286) of students had multiple sexual partners during the 12 months leading to the date of the survey. Condom use during last sexual encounter was reported by 50% of the students.

**Table 2:** Levels of sexual behaviors among the respondents

Variable	Measurements	*N*	%
Intention to engage is safe sex			
	No	92	23
	Yes	311	77
Age at sex debut			
	old > 13 yrs	354	88
	young ≤13 yrs	49	12
Number of sexual partners in the past 12 months			
	had one partner	117	29
	had multiple partners	286	71
Condom use during last sexual encounter (*n*=)^2^			
	Used a condom	78	50
	Did not use a condom	77	50

^2^Please note that there were 155 students who had started having sex by the date of the study

Table 3 presents the bivariate analysis results between the explanatory variables and sexual behaviours. The table displays results from the chi-square tests of independence between the sexual behaviors and social cohesion variables. The table further presents the same test with control variables age, religion and sex of the students. It can be seen that young age at sexual debut was independent of either social participation at school (*p* = 0.32) or social participation at the community level (*p* = 0.63). In addition, having multiple sexual partners was also independent of either social participation at school (*p* = 0.60) or social participation at the community level (*p* = 0.20).

**Table 3:** Bivariate relationship between explanatory variables and sexual behaviours

Variable		Age at sexual debut (young ≤ 13 yrs)		Multiple sexual partners (> 1 partner)		No intention to use condom		No condom use	
		n (%)	*p*-value	n (%)	*p*-value	n (%)	*p*-value	n (%)	*p*-value
Social participation at school (*n* = 247)^1^									
	Weak participation	8 (15)	0.32	7 (27)	0.6	18 (75)	0.07	12 (23)	0.55
	2	5 (10)		3 (15)		9 (47)		12 (24)	
	3	3 (6)		4 (29)		8 (62)		7 (14)	
	4	14 (18)		13 (34)		16 (44)		14 (18)	
	Strongest participation	1 (8)		2 (40)		1 (20)		4 (31)	
Social participation at the community (n = 403)									
	Weak participation	12 (10)	0.63	12 (31)	0.2	16 (44)	0.76	31 (25)	0.85
	2	6 (15)		3 (21)		7 (58)		8 (20)	
	3	9 (11)		5 (15)		15 (50)		17 (21)	
	4	10 (11)		13 (30)		25 (57)		18 (20)	
	Strongest participation	12 (17)		14 (41)		15 (45)		18 (25)	
Age (*n* = 403)									
	14–16 years old	16 (13)	0.86	3 (12)	0.045	12 (50)	0.97	33 (26)	0.31
	17–19 years old	33 (12)		44 (32)		50.38 (0)		59 (21)	
Religion (*n* = 403)									
	Non Muslim	7 (13)	0.76	6 (27)	0.88	13 (65)	0.16	10 (19)	0.51
	Muslim	42 (12)		41 (29)		65 (48)		82 (23)	
Sex (*n* = 403)									
	Male	37 (18)	< 0.001	32 (34)	0.08	42 (47)	0.28	53 (26)	0.11
	Female	12 (6)		15 (21)		36 (55)		39 (20)	

For the control variables, age at sexual debut was independent of the age or religion of the student. However, age at sexual debut was dependent on sex of the student (*p* < 0.001), and having multiple sexual partners was dependent on the age (*p* = 0.045) of the student.

Table 4 presents the results of a regression analysis that was conducted to assess how sexual behaviors are associated with social cohesion. The age at sexual debut was not found to be associated with the level of social participation, as was predicted by the bivariate analysis presented earlier. Age at sexual debut was, however, associated with the controlling variable sex where being female (OR = 0.21 95% CI = 0.07–0.57) was associated with having a decreased likelihood of having one’s sexual debut at a young (≤13 years of age). The number of sexual partners was also not found to be associated with the level of social participation but was associated with medium social trust (OR = 3.52, 95% 1.01–12.30). There was also no statistically significant association found between multiple sexual partnership and the controlling variables. Being female (OR = 2.07, 95% CI =1.04–4.12) was associated with intention to use condoms, and the use of condoms was found to be associated with medium social cohesion (OR = 4.83, 95% CI =1.66–14.06).

**Table 4:** Logistic regression model for explanatory variables and sexual behaviors

Variable	Age at sexual debut (young ≤13 yrs)		Multiple sexual partners (> 1 partner		Intention to use condom		Reported condom use	
	OR (95% CI)	*p*-value	OR(95% CI)	*p*-value	OR(95% CI)	*p*-value	OR(95% CI)	*p*-value
Social cohesion								
Low social cohesion	Reference		Reference		Reference		Reference	
Medium social cohesion	0.51 (0.19–1.33)	0.17	0.87 (0.28–2.65)	0.8	1.87 (0.85–4.11)	0.11	4.83 (1.66–14.06)	0.00
High social cohesion	1.86 (0.62–5.57)	0.26	2.36 (0.66–8.37)	0.18	1.16 (0.47–2.83)	0.73	2.31 (0.70–7.63)	
Social trust								
Low social trust	Reference		Reference		Reference		Reference	
Medium social trust	2.6 (0.94–7.17)	0.06	3.52 (1.01–12.30)	0.04	1.42 (0.59–3.39)	0.42	0.98 (0.29–3.24)	0.98
High social trust	1.71 (0.58–4.98)	0.32	1.98 (0.59–6.69)	0.26	0.64 (0.30–1.35)	0.24	1.251 (0.38–4.03)	0.71
Social participation								
Low social participation	Reference		Reference		Reference		Reference	
Medium social participation	0.81 (0.26–2.46)	0.7	0.73 (0.19–2.76)	0.64	2.73 (0.92–8.10)	0.06	0.367 (0.10–1.33)	0.12
High social participation	0.55 (0.20–1.47)	0.23	1.15 (0.35–3.80)	0.81	1.16 (0.55–2.45)	0.68	1.42 (0.45–4.41)	0.54
Age								
14–16 years	Reference		Reference		Reference		Reference	
17–19 years	0.76 (0.33–1.75)	0.52	5.06 (0.83–30.74)	0.07	1.98 (0.94–4.17)	0.07	1.42 (0.37–5.41)	0.16
Sex								
Male	Reference		Reference		Reference		Reference	
Female	0.21 (0.07–0.57)	0.002	0.61 (0.22–1.72)	0.35	2.07 (1.04–4.12)	0.03	0.44 (0.17–1.11)	0.08
Religion								
Non-Muslim	Reference		Reference		Reference		Reference	
Muslim	0.99 (0.28–3.44)	0.99	1.21 (0.19–7.59)	0.83	0.68 (0.24–1.92)	0.47	3.07 (0.72–3.04)	0.12

## Discussion

The purpose of the present study was to determine the proportion of adolescents who practice safe sex and to establish the influence of social cohesion, and its components (social trust and social participation) variables, on the safe sexual behaviors. The study found that the majority of adolescents in the selected sample became sexually active at an age older than 13 years, thus supporting the existing evidence in the country, and elsewhere, on the changing trends on age at first sex [26, 39, 40]. The results showing that females do not start sex earlier is a new finding and may need further exploration in the line of understanding the power of social cohesion to influence sexual behaviours, as existing evidence indicates that girls start sex earlier than boys [40]. Also, the majority of sexually active adolescents in our sample had multiple partners, which is in-line with previous studies that have indicated multiple sexual partnerships as being one of the persistent challenges in fighting the spread of sexually transmitted infections and HIV infection in the Sub-Saharan Africa settings [3, 6]. The study also found that approximately half of the studied sample reported to have used condoms during last sexual encounter. The number of adolescents reporting the use of condoms during their last sexual encounters has been described by other studies to be increasing, in some studies reaching as high as 70 % of the respondents [3, 10, 41].

The finding that adolescents participated more in community based activities than those at school may imply that more opportunities are created at community level to enhance openness and eagerness of adolescents to participate in safe sex promoting activities. Given the multiplicity of actors engaged in scaling up HIV/ AIDS interventions to enhance universal access, several community based activities that include formation of civil society organizations and peer education groups have been implemented in these settings, thus creating an environment that supports social cohesion. Also, the fact that the studied population was predominantly of the same religion, makes it a favourable environment for social networking created by the context. Bramadt assets that religion sustains social cohesion and answers personal needs, thus creating a positive social networking environment in the given context [42]. But this assertion may need further inquiry especially in other settings with mixture of religious beliefs.

We found that social trust was associated (though not statistically significant) with multiple sexual partners. The finding may imply that adolescents who display social trust to significant others may end up practicing a behavior that looks to be appealing to the group the individual shows trust. Exploring further on the link between social trust and multiple sexual partnerships is important but also designing interventions that penetrate social networks, such as behavior change interventions targeting the youth groups, and influential significant others at school (teachers and peer leaders) and community level (parents, influential community and religious leaders), may help to entangle the complexity of multiple sex partnerships in these settings.

We also found that none of the social cohesion variables had significant influence on intentions to use a condom. This finding may imply that intentions may be influenced by individual level factors such as social demographics, socio-cognitive, and other related factors, as displayed in this study that one’s gender (being a female) whereas other societal and structural factors may be related to actual behavior performance.

Reported condom use was found to be associated with social cohesion. A recent review [4] on condom use among adolescents of sub-Saharan Africa supports the view that wider societal and structural influences are determinants of health behaviors, including condom use, and they explain that much of the existing evidence has been on individual level factors. The fact that may be limiting and not providing a clear picture of the influence of social-ecological factors on sexual behaviors. This may imply that future studies need to focus on influence of socio-ecological determinants in different settings.

In this study, the social cohesion variables are assumed to act on behaviors directly or via intentions (for condom use). Evidence has attested to the fact that intentions are strong predictors of actual behavior performance [31]. Informed by previous research on sexual behaviors, we postulated that social cohesion variables influence sexual behaviors different from individual factors (under individual control) that may follow conscious or volition control [26, 31].

The strength of the study is that social cohesion variables displayed at both the community and school environments were investigated. Being that the study was conducted using a cross-sectional inquiry, and only involved a small sample of one district of the country; the findings in this should only be interpreted within its contexts. Moreover, since there are no major changes in the study settings from the time the data was collected to the time this manuscript is submitted for publication, the observations made in the study should remain relevant.

## Conclusion

The results of this study have highlighted the important social cohesion variables that have shown to be associated with sexual behaviors of adolescents. Importantly, social trust was shown to have an influence on the number of sexual partners in adolescents in SSA, whereas, social cohesion has influence on reported condom use but no significant influence on the intention of condom use. The results of the study showed that social participation has no influence on sexual behaviors. The lack of current literature suggests that further studies on the influence of social cohesion variables on sexual behavior, in different settings or similar settings, using more rigorous study designs, are needed. Furthermore, interventions that target social cohesion may be helpful in the attempt to change sexual behavior of adolescents, and could be valuable in guiding future policy directions regarding adolescent sexual behaviors.

## Additional file

Additional file 1: Questionnaire for school adolescents. (DOCX 36 kb)

## Abbreviations

AIDS: Acquired Immunodeficiency Syndrome
CI: Confidence interval
CSO: Civil Society organization
HIV: Human Immunodeficiency virus
IFP: International Fellowship program
MCA: Multiple correspondence analysis
OR: Odds ratio
PCA: Principal component analysis
SSA: Sub-Saharan Africa
