# Supplementary material for: Social cohesion, social trust, social participation and sexual behaviors of adolescents in rural Tanzania
Source: BMC Public Health. 2019 Feb 14;19:193. doi: 10.1186/s12889-019-6428-7 (PMC6376705; doi:10.1186/s12889-019-6428-7)
Supplement: Supplementary file 1 — Questionnaire for school adolescents. (DOCX 36 kb) [file 12889_2019_6428_MOESM1_ESM.docx]

## Additional file 1: Questionnaire for school adolescents

**SOCIAL COHESION AND SEXUAL BEHAVIORS OF ADOLESCENTS IN RURAL TANZANIA**

| **QUESTIONNAIRE FOR SCHOOL ADOLESCENTS** |
| --- |

**BACKGROUND INFORMATION**

| Questionnaire number |  |
| --- | --- |
| Date of an Interview |  |
| Name of school |  |
| Form/ Class |  |

***Fill or mark √ in the box for the answer in the following questions***

**SECTION 1: SOCIO-DEMOGRAPHIC INFORMATION**

| No | Question | Response |
| --- | --- | --- |
| 1 | What is your sex? | 1. Male 2. Female |
| 2 | What is your age ( years) | \|_____\|_____\| |
| 3 | What is your religion | 1. Moslem  2.Roman catholic  3.Anglican  4. Lutheran  5. Pentecost  6.Others (specify) |

**SECTION 2: - SOCIAL PARTICIPATION**

***Mark √ in the box for the answer of your choice in the following questions***

| 4 | Do you participate in the following activities | Yes | No |
| --- | --- | --- | --- |
| 4.1 | Peer education |  |  |
| 4.2 | Music competition with safe sex promotion /HIV prevention themes |  |  |
| 4.3 | Life skill training on sexual and reproductive health |  |  |
| 4.4 | Sports bonanza with safe sex promotion themes |  |  |

| 5 | Are you a member of | Yes | No |
| --- | --- | --- | --- |
| 5.1 | Youth NGO dealing with safe sex promotion /HIV prevention among other issues |  |  |
| 5.2 | Youth group dealing with safe sex promotion /HIV prevention at school or at the community |  |  |
| 5.3 | Youth club/camp dealing safe sex promotion /HIV prevention |  |  |
| 5.4 | Youth sports group dealing with safe sex promotion /HIV prevention |  |  |
| 5.5 | Group of People living with HIV/AIDS |  |  |
| 5.6 | Arts group or traditional dance group dealing with safe sex promotion /HIV prevention |  |  |

|  |  | Yes | No |
| --- | --- | --- | --- |
| 6 | Does your school provide training on life skills for HIV/AIDS prevention? |  |  |

(If No go to 8)

| 7 | How do you participate in the these trainings | Yes | No |
| --- | --- | --- | --- |
| 7.1 | Planning |  |  |
| 7.2 | Facilitating |  |  |
| 7.3 | Playing a role play |  |  |
| 7.4 | As a role model |  |  |
| 7.5 | Listening only |  |  |

SECTION 3: SOCIAL TRUST

***The following questions concerns personal opinion on various matters about sexual practices (to strong agree, agree, uncertain, disagree, or strongly disagree). There is no right or wrong answer; it depends on individual’s opinion. We would like to know your opinion. Choose on one answer in the box and mark √ in the respective box***

|  |  | Agree | Uncertain | Disagree |
| --- | --- | --- | --- | --- |
| 8 | My parents/guardians think that I should not have sexual intercourse before marriage |  |  |  |
| 9 | I agree with my parents/guardians opinion that I should not have sexual intercourse before marriage |  |  |  |
| 10 | My friends think that I should use condoms when having sexual intercourse |  |  |  |
| 11 | I agree with the opinion of my friends that I should use condoms when having sex |  |  |  |
| 12 | My teachers think that I should abstain from sex until I get married |  |  |  |
| 13 | I agree with my teachers opinion that I should not have sex until marriage |  |  |  |
| 14 | My friends think that I should have a boy/girlfriend to show that I am physically fit |  |  |  |
| 15 | I agree with my friends’ opinion that I should have a boy/girlfriend to show that I am physically fit |  |  |  |
| 16 | My friends think that having more than one sexual partner is more appealing for a youth |  |  |  |
| 17 | I agree with my friends’ opinion that having more than one sexual partner is appealing |  |  |  |
| 18 | My religious leaders think that I should not use condoms when having sex |  |  |  |
| 19 | I agree with my religious leaders opinion that I should not use condoms when having sexual intercourse |  |  |  |

**SECTION 4: SEXUAL BEHAVIOURS**

| No | Question | Response |
| --- | --- | --- |
| 20 | Have you ever had penetrative sexual intercourse?... if No, go to 25.. | 1. Yes 2. No |
| 21 | At which age did you start having penetrative sexual intercourse ( years) | \|_____\|_____\| |
| 22 | How many sexual partners have you had in your life? | \|_____\|_____\| |
| 23 | How many sexual partners have you had in last 12 months?. | \|_____\|_____\| |
| 24 | Did you use a condom at your last penetrative sexual intercourse? | 1. Yes 2. No |
| 25 | Do you intend to use a condom next time you have penetrative sexual intercourse? | 1. Yes 2. No |
